# Bronchiectasis and airspace enlargement surrounding the lung nodule in dual-energy CT pulmonary angiography: comparison between iodine map and monochromatic image

**DOI:** 10.1007/s12194-025-00920-3

**Published:** 2025-06-05

**Authors:** Koichiro Yasaka, Jun Kanzawa, Shohei Inui, Takatoshi Kubo, Osamu Abe

**Affiliations:** Department of Radiology, The University of Tokyo Hospital, 7-3-1 Hongo, Bunkyo-Ku, Tokyo, 113-8655 Japan

**Keywords:** Multidetector computed tomography, Lung cancer, Lung nodule, Bronchiectasis

## Abstract

The purpose of the study is to investigate the degree and performance in the differential diagnosis of bronchiectasis/airspace enlargement in an iodine map obtainable from CT pulmonary angiography compared with monochromatic images. This retrospective study included 62 patients with a lung nodule who underwent CT pulmonary angiography. The iodine map and monochromatic image (70 keV) were reconstructed. Three readers evaluated the degree of bronchiectasis/airspace enlargement with a 4-point scale. A reference standard was established in 39 patients, and the performance of bronchiectasis/airspace enlargement in the differential diagnosis was evaluated in them. The degree of bronchiectasis/airspace enlargement in the iodine map (median score = 1/2/1 for reader 1/2/3) was significantly more prominent than that in the monochromatic image (median score = 0/1/0 for reader 1/2/3) (*p* < 0.001 for all readers). Using bronchiectasis/airspace enlargement, primary lung carcinoma and malignant lymphoma could be differentiated from other diseases, excluding lung infarct, with an area under the receiver operating characteristic curve (AUC) (reader 1/2/3) of 0.718/0.867/0.803 in the combinations of iodine map plus monochromatic image and 0.496/0.828/0.450 in the monochromatic image (*p* ≤ 0.047 for two readers). Lung metastasis from colorectal carcinoma could be differentiated from other diseases with an AUC of 0.851/0.976/0.838 in the combinations of iodine map plus monochromatic image, which was significantly superior to the monochromatic image (0.378/0.780/0.459) (*p* ≤ 0.012 for all readers). Bronchiectasis/airspace enlargement was more prominently observed in the iodine map than in the monochromatic image. This image finding in the iodine map provided added value in the differential diagnosis of malignant lung nodules compared with monochromatic images alone.

## Introduction

Computed tomography (CT) is typically utilized to detect lung nodules. According to Swensen, 51% of asymptomatic participants aged over 50 years and who had at least a 20 pack-year smoking history had one or more lung nodules [1]. Although lung nodules can be benign, such as intrapulmonary lymph nodes and old tuberculosis, some can be malignant. Considering that one of the most frequently diagnosed carcinomas and the leading cause of cancer-related deaths is primary lung carcinoma [2] and that the lung is one of the most prominent target organs for metastatic disease [3], a differential diagnosis of lung nodules is clinically important.

Several articles have been published regarding the differential diagnosis of lung nodules. Recent studies have employed newly developed techniques [4], such as radiomics [5], machine-learning [6], deep learning [7], and dual-energy CT [8–10]. Despite their high performance, radiomics and machine-learning techniques require complicated analyses, which hinders their application in busy daily clinical practice. Deep learning is also a promising technique; however, it requires a large amount of data in training the model to achieve high performance [4, 7, 11].

There are several types of applications for dual-energy CT, such as virtual nonenhanced images and iodine maps. While it has been reported that malignant nodules are enhanced by 20 Hounsfield units or greater [12], scanning of both enhanced and unenhanced CT images requires increased radiation exposure. Chae et al. reported that virtual nonenhanced images were useful in estimating the degree of lung nodule enhancement without additional radiation exposure [13]. Quantitatively evaluated normalized iodine concentration and the slope of the spectral Hounsfield unit curve demonstrated superior performance in delineating malignant nodules from benign ones compared with conventional CT attenuation enhancement [14]. While those reports focused on the internal characteristics of lung nodules, imaging features surrounding lung nodules (such as spiculation [15, 16], pleural indentation [16, 17], etc.) also play important roles in the differential diagnosis of them. In addition, we serendipitously discovered that bronchiectasis and airspace enlargement are more prominently depicted in the iodine map compared with the monochromatic image, which has not reported before. Because malignant lung nodules are more likely to cause morphological changes surrounding them as described above, we hypothesized that this image finding observable in the iodine map also have a potential to contribute to the differential diagnosis of lung nodules.

This study aimed to compare the degree of bronchiectasis/airspace enlargement surrounding the lung nodules between iodine maps and monochromatic images and to assess its performance in the differential diagnoses.

## Materials and methods

This retrospective study was approved by our institutional review board, which waived the requirement for obtaining written informed consent from patients owing to the retrospective nature of this study.

### Patients

**Fig. 1:**
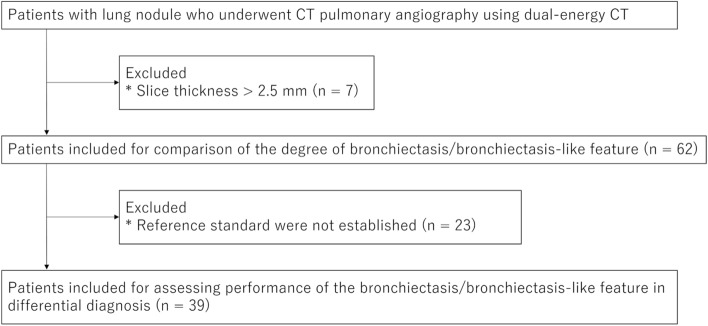
Flowchart of the patient inclusion process

We searched picture archiving and communication systems for patients with lung nodules who underwent dual-energy CT pulmonary angiography to evaluate pulmonary embolisms from October 2019 to February 2023. Patients whose images had a slice thickness of more than 2.5 mm were excluded from this study (*n* = 7) (Fig. 1).

### CT imaging

All patients underwent CT examination using a CT scanner (Revolution CT [GE Healthcare, WI, US]). Iodine contrast material (600 mgI/kg) (iomeron [Bracco], iopamiron [Bayer], omnipaque [GE Healthcare], or optiray [Guerbet]) was injected from the antecubital vein within 30 s using an automatic power injector. The scan was performed 20 s after initiating the contrast material injection. The iodine concentration of the contrast material was determined based on the body weight: 320, 350, and 370 mgI/mL for <48, 49–63, and >64 kg, respectively. The scanning parameters were as follows: tube voltage, fast kVp switching with 80 kVp and 140 kVp; tube current, auto mA with noise index of 11.4 (setting slice thickness of 5 mm); gantry rotation time, 0.5 s; pitch factor, 0.992. From the source data, the iodine map (with the projection-based method using iodine and water as the reference material) and 70-keV monochromatic image (filtered back projection) were reconstructed. CT attenuation of the 70-keV monochromatic image corresponds to that of 120-kVp CT image [18]. Slice thickness was 1.25 or 2.5 mm, which was the same between the iodine map and monochromatic image within a patient. The number of patients whose images were reconstructed with a slice thickness of 1.25 mm and 2.5 mm was 31 and 31, respectively. Field of view/pixel size for the iodine map and monochromatic image were 350–450 mm/0.684–0.879 mm and 200–240 mm/0.391–0.469 mm, respectively.

### Image evaluation

In the subjective image evaluation, three radiologists (readers 1, 2, and 3 with imaging experience of 11, 7, and 2 years, respectively) were involved. They were blinded to patient background and nodule diagnostic information. Default window level/width for iodine maps and monochromatic images were 15/110 mgI/ml and −600/1600 Hounsfield unit, respectively. However, as in daily clinical practice, readers were allowed to adjust these settings according to their preferences. In addition, they were allowed to magnify images during observation. They independently evaluated the degree of bronchiectasis/airspace enlargement surrounding the lung nodule using a 4-point Likert scale (2 = bronchiectasis/airspace enlargement is prominent compared to normal lung parenchyma at a similar distance from the lung hilum, 1 = bronchiectasis/airspace enlargement is slightly greater than normal lung parenchyma at a similar distance from the lung hilum, 0 = bronchial diameter is the same degree to the normal lung parenchyma at a similar distance from the lung hilum, −1 = stricture of bronchus is present). To assess the added value of the degree of bronchiectasis/airspace enlargement on the iodine map, a logistic regression model was constructed using the scores from both the monochromatic image and the iodine map. The lung nodule to be evaluated was annotated by another radiologist A (with imaging experience of 14 years). In cases where several lung nodules were present, the most representative one was evaluated. Prior to the above image evaluation, radiologist A randomized all image sets into two parts, ensuring that a single patient image set was present in each part. There was a 2-week interval between parts 1 and 2. Additionally, to evaluate the intraobserver agreement, radiologist A randomly selected 15 patients, and their images were evaluated by the three readers again in part 3 after a 2-week interval from part 2.

### Reference standard

A reference standard could be established for 39 patients. Among them, 14 patients were diagnosed histopathologically (adenocarcinoma [*n* = 5], squamous cell carcinoma [*n* = 3], small cell lung carcinoma [*n* = 3], malignant lymphoma [*n* = 2], and organizing pneumonia [*n* = 1]). Lung nodules were diagnosed as metastasis in 14 patients with a combination of past history of malignancy and enlargement from past CT examination (colorectal carcinoma [*n* = 4], cervical carcinoma [*n* = 1], endometrial carcinoma [*n* = 3], uterine sarcoma [*n* = 1], cholangiocellular carcinoma [*n* = 1], lung carcinoma in other location [*n* = 2], malignant melanoma [*n* = 1], and chordoma [*n* = 1]). Lung nodules that later decreased in size or diminished were considered as inflammation (*n* = 5). Lung nodules in patients with proximal pulmonary embolism were diagnosed as lung infarcts (*n* = 2). Lung nodules in patients with a history of old tuberculosis and nontuberculous mycobacterium were diagnosed as granulomas (*n* = 3). Lung nodules with angular margins were diagnosed as intrapulmonary lymph nodes (*n* = 1).

### Statistical analysis

Statistical analyses were performed using R version 4.1.2. Scores were compared between the iodine map and monochromatic image using the Wilcoxon signed rank test. To estimate the performance using the degree of bronchiectasis/airspace enlargement in the differential diagnoses, the area under the receiver operating characteristic curve (AUC) was calculated and compared between a combination of iodine map plus monochromatic image (logistic regression model) *vs.* monochromatic image alone using the DeLong test. A *p* value of less than 0.050 was considered indicating a statistically significant difference. To evaluate the intra- and interobserver agreement, Cohen’s kappa values were calculated.

## Results

### Patients

Patient background information is detailed in Table 1. In this study, 62 patients (mean age, 67.8 ± 13.2 years; 32 males and 30 females) were included. Among them, there were 39 patients (mean age, 67.9 ± 11.2 years; 15 males and 18 females) for whom the reference standard could be established.

**Table 1:** Background information of patients

Category	Value
Age (years)	67.8 ± 13.2
Sex (male/female)	32/30
Lung nodule main location (*n*)	
Right upper lobe	17
Right middle lobe	10
Right lower lobe	17
Left upper lobe	13
Left lower lobe	5
Lung nodule size (mm)	17.2 ± 20.2
Lung nodule internal characteristics (*n*)	
Solid	51
Ground glass	4
Cavity	4
Calcification	3
Lung nodule margin (*n*)	
Regular	33
Irregular	29

### Bronchiectasis/airspace enlargement in each image

**Fig. 2:**
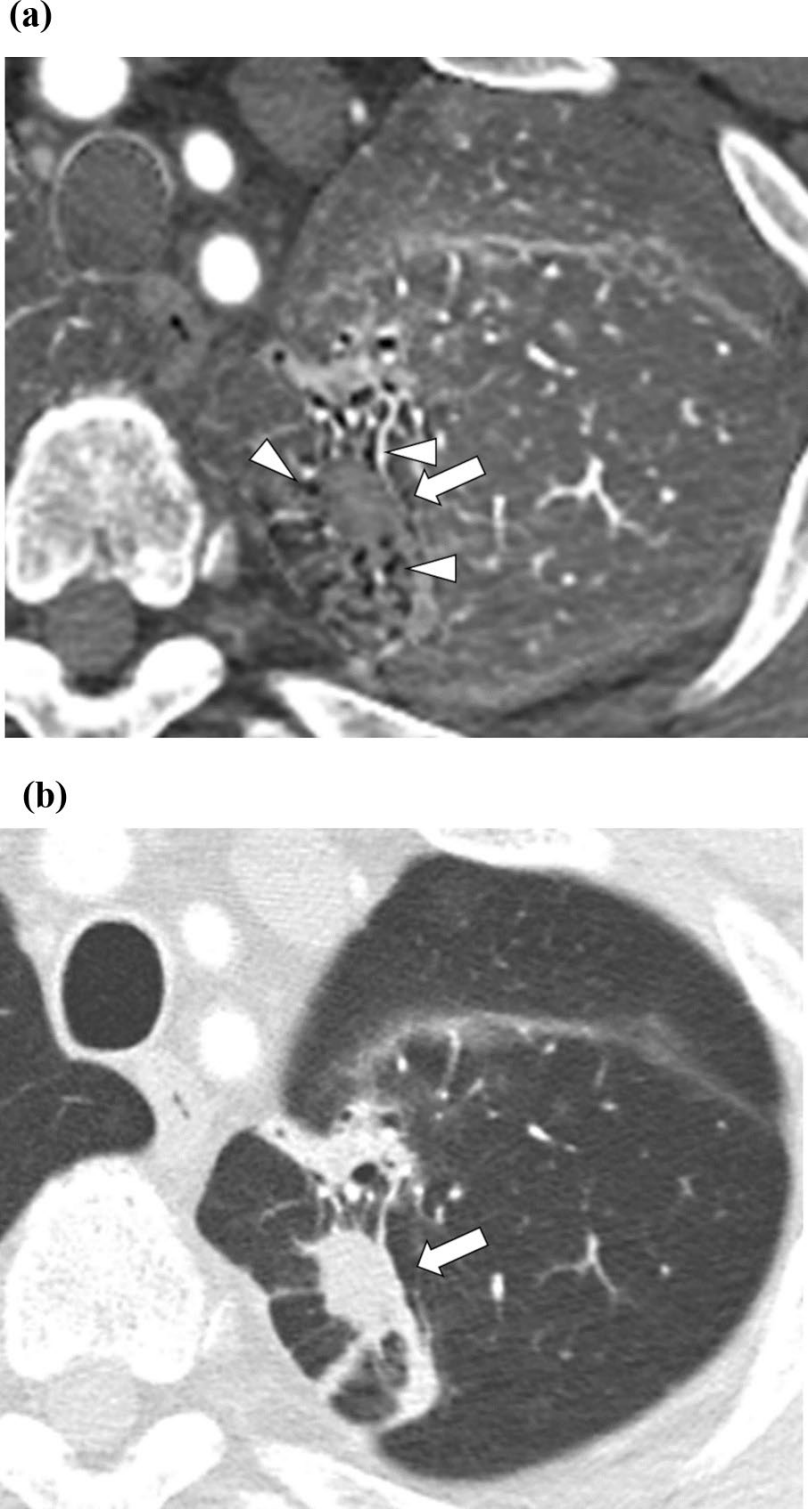
Iodine map and monochromatic image of a 74-year-old male patient with a primary lung squamous cell carcinoma (white arrows). Readers 1/2/3 rated the degree of bronchiectasis/airspace enlargement (arrowheads) surrounding the nodule as 2 (prominent)/2/2 and 0 (the same degree)/2/0 for the iodine map (**a**) and monochromatic image (**b**), respectively

**Fig. 3:**
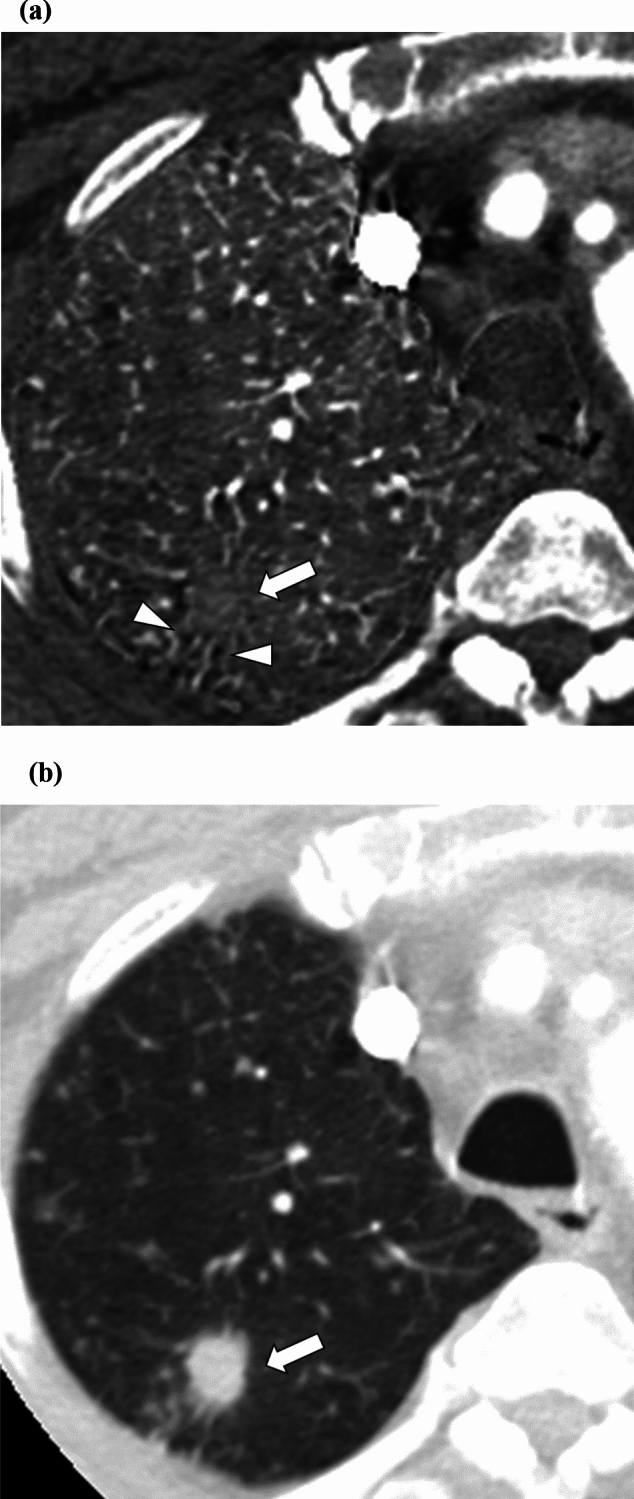
Iodine map and monochromatic image of a 65-year-old male patient with a primary lung adenocarcinoma (white arrows). Readers 1, 2, and 3 rated the degree of bronchiectasis/airspace enlargement (arrowheads) surrounding the nodule as 2 (prominent), 2, and 2 and 0 (the same degree), 1 (slightly greater), and 0 for the iodine map (**a**) and monochromatic image (**b**), respectively

The scores for bronchiectasis/airspace enlargement by the three readers are demonstrated in Table 2. All readers agreed that the degree of bronchiectasis/airspace enlargement was significantly more prominent in the iodine map (readers 1, 2, and 3 scored 2/1/0/-1 for 24/31/7/0, 43/10/9/0, and 23/10/28/1 patients, respectively) than in the monochromatic image (readers 1, 2, and 3 scored 2/1/0/-1 for 1/17/44/0, 18/20/24/0, and 3/6/49/4 patients, respectively) (*p* < 0.001). A representative iodine map and monochromatic image are shown in Figs. 2 and 3.

**Table 2:** Scores for the degree of bronchiectasis/airspace enlargement

Reader	Iodine map	Monochromatic image	Comparison
1	24/31/7/0	1/17/44/0	<0.001*
2	43/10/9/0	18/20/24/0	<0.001*
3	23/10/28/1	3/6/49/4	<0.001*

For the iodine map and monochromatic image columns, the numbers of patients for scores 2 (prominent), 1 (slightly greater), 0 (the same degree), and −1 (stricture) are presented

* Statistically significant difference (*p* < 0.050)

### Distribution of bronchiectasis/airspace enlargement in each disease entity

**Fig. 4:**
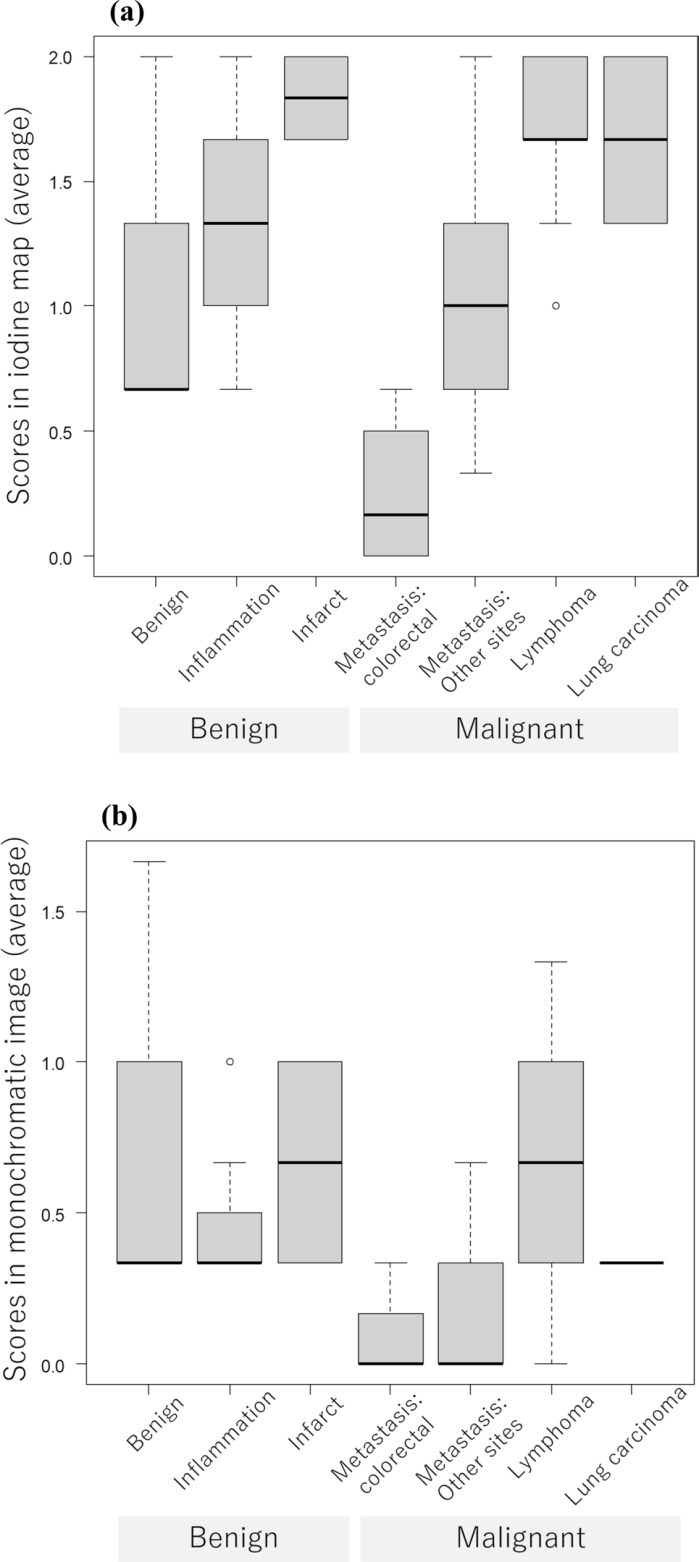
Box and whisker plots for the averaged bronchiectasis/airspace enlargement scores *vs.* diseases in the iodine map (**a**) and monochromatic image (**b**)

The distribution of readers’ scores for bronchiectasis/airspace enlargement is revealed in Fig. 4. The median for the averaged bronchiectasis/airspace enlargement score for benign, inflammation, infarct, metastasis from colorectal carcinoma, metastasis from other sites, malignant lymphoma, and primary lung carcinoma was 0.67, 1.33, 1.83, 0.17, 1.00, 1.67, and 1.67, respectively in the iodine map and 0.33, 0.33, 0.67, 0.00, 0.00, 0.67, and 0.33, respectively in the monochromatic image.

### Diagnostic performance

**Fig. 5:**
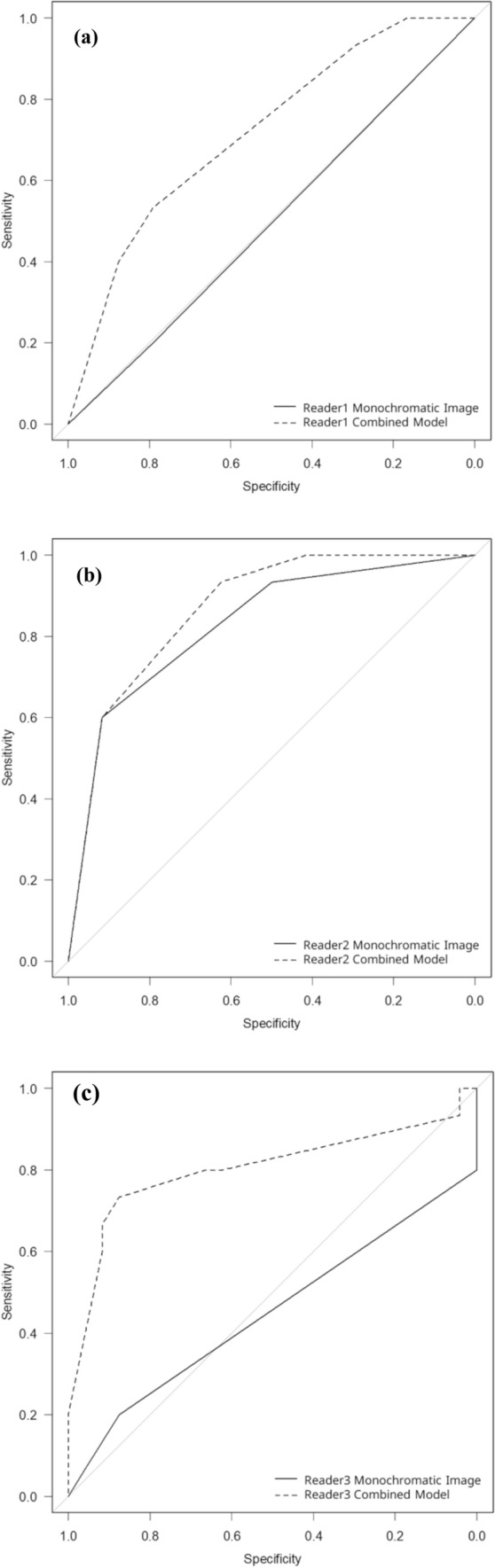
Receiver operating characteristic curves in differentiating primary lung carcinoma and malignant lymphoma from other diseases excluding lung infarct by readers 1 (**a**), 2 (**b**), and 3 (**c**). Dotted and solid lines indicate curves for the combined model (iodine map plus monochromatic image) and monochromatic image, respectively

Using bronchiectasis/airspace enlargement, primary lung carcinoma and malignant lymphoma could be differentiated from other diseases, excluding lung infarct (Fig. 5), with an AUC (reader 1/2/3) of 0.718/0.867/0.803 in the combined model (iodine map plus monochromatic image) and 0.496/0.828/0.450 in the monochromatic image (Table 3). Statistically significant differences were found in the AUC between the iodine map and monochromatic image for readers 1 and 3 (*p* = 0.047 and 0.011, respectively). Although AUC in the combined model tended to be superior to that in the monochromatic image for reader 2, no statistically significant difference was observed between them (*p* = 0.106). The sensitivity/specificity in the combined model were 0.533/0.792, 0.933/0.625, and 0.733/0.875 for reader 1, 2, and 3, respectively and those in monochromatic image were 0.200/0.792, 0.600/0.917, and 0.200/0.875 for reader 1, 2, and 3, respectively.

**Table 3:** Diagnostic performance results

	Monochromatic image	Combined model	Comparison
Differentiation of primary lung carcinoma and malignant lymphoma			
Reader 1	0.496	0.718	0.047*
Reader 2	0.828	0.867	0.106
Reader 3	0.450	0.803	0.011*
Differentiation of lung metastasis from colorectal carcinoma			
Reader 1	0.378	0.851	<0.001*
Reader 2	0.780	0.976	0.012*
Reader 3	0.459	0.838	<0.001*

Areas under the receiver operating characteristic curve are presented

* statistically significant difference (*p* < 0.050)

**Fig. 6:**
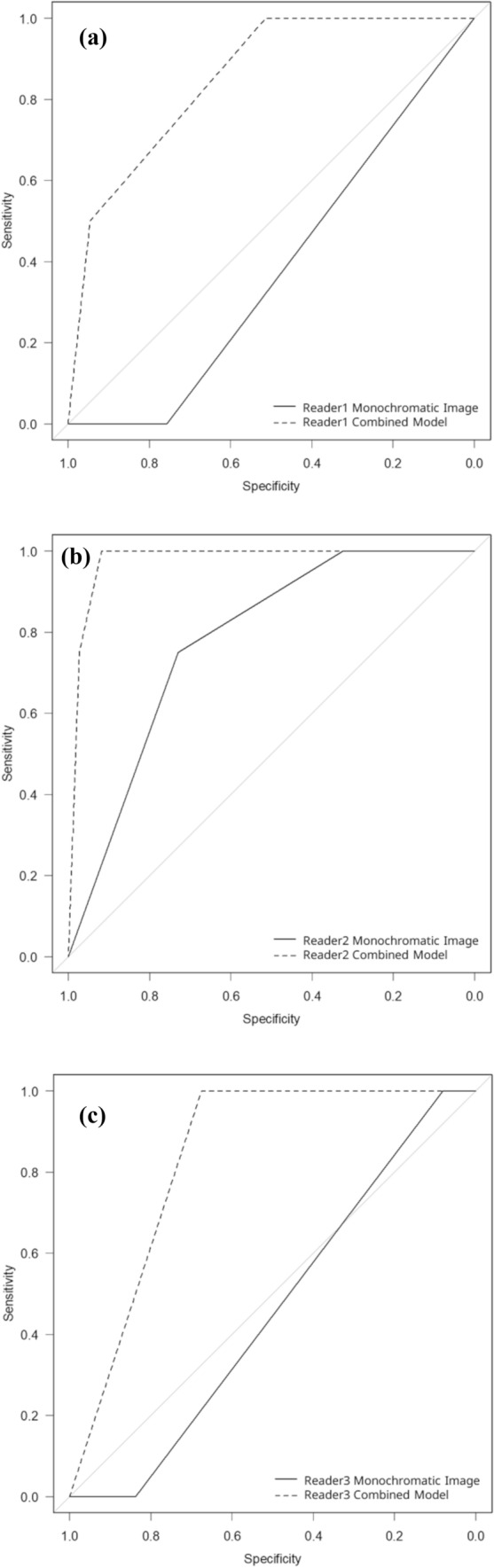
Receiver operating characteristic curves in differentiating lung metastasis from colorectal carcinoma from other diseases by readers 1 (**a**), 2 (**b**), and 3 (**c**). Dotted and solid lines indicate curves for the combined model (iodine map plus monochromatic image) and monochromatic image, respectively

In differentiating lung metastasis from colorectal carcinoma from other diseases (Fig. 6), the bronchiectasis/airspace enlargement showed an AUC (reader 1/2/3) of 0.851/0.976/0.838 in the combined model, which was significantly higher than that in the monochromatic image for all readers (0.378/0.780/0.459) (*p* ≤ 0.012) (Table 3). The sensitivity/specificity in the combined model were 1.000/0.514, 1.000/0.919, and 1.000/0.676 for reader 1, 2, and 3, respectively and those in monochromatic image were 1.000/0.000, 0.730/0.750, and 1.000/0.081 for reader 1, 2, and 3, respectively.

### Intra- and interobserver agreement

The results for intra- and interobserver agreement are illustrated in Table 4. Both the intra- and interobserver agreement in the iodine map (0.477–0.590 and 0.409–0.456, respectively) were comparable to those in the monochromatic images (0.423–0.571 and 0.287–0.438, respectively).

**Table 4:** Intra- and interobserver agreement in the evaluations of bronchiectasis/airspace enlargement

	Iodine map	Monochromatic image
Intra-observer agreement		
Reader 1	0.590 (0.296–0.884)	0.571 (0.211–0.932)
Reader 2	0.477 (0.152–0.802)	0.511 (0.207–0.815)
Reader 3	0.551 (0.229–0.872)	0.423 (-0.062–0.908)
Inter-observer agreement		
Reader 1 *vs.* 2	0.431 (0.285–0.578)	0.287 (0.159–0.414)
Reader 2 *vs.* 3	0.409 (0.261–0.556)	0.369 (0.226–0.512)
Reader 3 *vs.* 1	0.456 (0.316–0.597)	0.438 (0.256–0.621)

In the parentheses, 95% confidence interval is provided

## Discussion

While there have been several reports that evaluated the utility of dual-energy CT in the differential diagnosis of lung nodules [12–14], the potential of bronchiectasis/airspace enlargement found in the iodine map obtainable from CT pulmonary angiography remained unclear. From our study, it became evident that bronchiectasis/airspace enlargement was more prominently observed in the iodine map than in the monochromatic image. There was less overlap in the degree of bronchiectasis/airspace enlargement among different diseases in iodine map compared to monochromatic image, and this image feature in the iodine map was found to have added value in the differential diagnosis of lung nodules compared with monochromatic images alone.

In the differential diagnosis of lung nodules, spiculation is an important finding. This image finding is highly predictive of malignancy, with a positive predictive value of up to 90% [15, 16]. According to Zwirewich et al., this is caused by a desmoplastic response [17]. Such a response would also cause pleural indentation [16, 19] and pitfall sign [20]. These are associated with retraction of surrounding structures, which might have also caused the bronchiectasis/airspace enlargement found in our study. Our study revealed that bronchiectasis/airspace enlargement was more prominently observed in the iodine map than in the monochromatic image. This might have been caused by the following reasons. One is that the iodine map was helpful in depicting the increased iodine concentration of the inflamed bronchial wall or desmoplastic site compared to the monochromatic image. Another possible reason is that the contrast agent does not distribute within the exudates in the bronchus or airway. While the presence of exudates results in higher CT attenuation on the monochromatic image, it does not increase iodine concentration, thereby enhancing the contrast between the bronchial wall and the exudates surrounding nodules. In addition, the relatively lower contrast between the lung nodule and background lung parenchyma on iodine map may have further clarified the depiction of this image finding. While the pixel size of iodine map is larger than that of monochromatic image, iodine map was significantly more useful in depicting the bronchiectasis and airspace enlargement surrounding the lung nodule compared to monochromatic image. This might have resulted from significantly better contrast resolution of iodine map.

Primary lung carcinoma and malignant lymphoma were associated with a greater degree of bronchiectasis/airspace enlargement in the iodine map. However, this tendency was not remarkable in the monochromatic images. This indicates the usefulness of the iodine map in the differential diagnosis of lung nodules. In fact, we found that primary lung carcinoma and malignant lymphoma can be differentiated from other diseases with an AUC = 0.718–0.867 in cases where the possibility of lung infarct was excluded by other imaging findings such as subpleural location and the presence of proximal pulmonary embolism. A statistically significant difference between the combined model and the monochromatic image was not observed for one of the readers. This might have been due to relatively high performance in using scores of the monochromatic image alone. For this reader, the specificity in the combined model was lower than that in the monochromatic image, likely due to differences in the curvature of the ROC curves near the upper-left region. Regarding lung metastasis from colorectal carcinoma, the degree of bronchiectasis/airspace enlargement was found to be low. This is in line with a previous study that reported that metastases from colorectal carcinoma have spiculated margins in only 6.3% of cases, whereas primary lung cancers have spiculated margins in 46.9% [21]. However, as it has also been reported that the margins are occasionally irregular and poorly defined for lung metastases from other origins [22], the degree of bronchiectasis/airspace enlargement was variable for lung metastases from other sites.

Since 2010s, research regarding the applications of radiomics to tumor imaging has been gaining wide attention. In radiomics studies, tumors are first segmented and quantitative features are extracted [5, 23–25]. Then, models, which are commonly based on machine-learning techniques such as support vector machine [26], random forest [27], and logistic regression [23, 24], are trained and validated. Despite the merits of the radiomics approach in several tasks, it is not devoid of demerits. The most serious one is that it requires tumor segmentation, which becomes an additional burden on busy daily clinical practice. On the contrary, bronchiectasis/airspace enlargement can be subjectively evaluated, and the results of our study would be immediately applicable to daily clinical practice.

This study has some limitations. First, the number of patients for whom a reference standard could be established was small. Because bronchiectasis/airspace enlargement in the iodine map was found to be more prominent than in the monochromatic image, future research involving larger numbers of patients for evaluating its usefulness in the differential diagnosis would be warranted. Second, because the design of this study was retrospective, selection bias may be present. Future prospective studies are necessary to consolidate the results of our study. Third, we used iodine map and monochromatic images generated from CT pulmonary angiography in this study. Because some disease entities are associated with increased vascularity, use of CT pulmonary angiography might have been one of the reasons for prominent bronchiectasis/airspace enlargement [28]. Whether this imaging feature is also depicted in iodine map generated from data acquired later than CT pulmonary angiography needs to be investigated in future studies. Fourth, iodine map changes with the amount of contrast material. It is reasonable to estimate that the bronchiectasis/airspace enlargement in iodine map correlates with the density and dose of iodine material. Therefore, our study results may not be directly applicable to patients who were administered different density or dose of iodine contrast material. Fifth, we did not include low keV-monochromatic images in the analyses, because they were not routinely reconstructed in our institution. It is expected that image findings of bronchiectasis/airspace enlargement would become more prominent in lower keV-monochromatic images. However, this needs to be validated in future studies. Sixth, because there were few patients with benign conditions, it was difficult to evaluate the diagnostic performance in distinguishing benign conditions from others. Finally, while there are different algorithms for dual-energy CT examination, we included images obtained using a single CT scanner in this study. Thus, the results of this study would not necessarily be directly applicable to dual-energy CT examinations performed by other vendors.

## Conclusion

In conclusion, bronchiectasis/airspace enlargement surrounding the lung nodule was more prominently observed in the iodine map than in the monochromatic image. This image finding in the iodine map provided added value for the differential diagnosis of malignant lung nodules compared to the monochromatic image alone.

## Data Availability

Due to the nature of this research, patients of this study did not agree for their data to be shared publicly, so supporting data is not available.
